# Automatic rib fracture detection on postmortem CT data using deep learning

**DOI:** 10.1007/s00414-025-03669-x

**Published:** 2025-12-04

**Authors:** Manel Lopez-Melia, Virginie Magnin, Sami Schranz, Vincent Andrearczyk, Adrien Depeursinge, Stéphane Marchand-Maillet, Silke Grabherr

**Affiliations:** 1 University Centre of Legal Medicine Lausanne-Geneva, University of Geneva, Rue Michel-Servet 1, 1206 Geneva, Switzerland; 2 Department of Computer Science, Viper Group, University of Geneva, Route de Drize 7, 1227 Carouge, Switzerland; 3 Department of Diagnostic and Interventional Radiology, University Hospital Lausanne, Lausanne, Switzerland; 4 Institute of Informatics, University of Applied Sciences Western Switzerland (HES-SO), Sierre, Switzerland; 5 Department of Nuclear Medicine and Molecular Imaging, University Hospital Lausanne, Lausanne, Switzerland

**Keywords:** Rib, Fracture, Detection, CT, Postmortem, Deep learning

## Abstract

**Objectives:**

To assess the performance of automatic rib fracture detection of an existing deep learning (DL) model, nnDetection, on a postmortem (PM) CT scan dataset, and to identify the main factors of domain shift between clinical and PM CT imaging.

**Background:**

Rib fracture detection and classification in forensic investigations is a time-consuming yet crucial task that can contribute to determine the cause of death. DL models are a promising tool, as recent research shows that radiologists using DL models can detect rib fractures in clinical CT scans at higher sensitivity and in shorter time.

**Methods:**

A dataset of 50 PMCT scans (24% women; age: mean 61, range 19 – 96 years) was retrospectively collected and annotated, and used to train a first instance of the model, nnDetPM. Another instance of the model, nnDetClin, was trained on data from another dataset, RibFrac, consisting of 660 clinical CT scans (36% women; age: mean 55, range 21 – 94 years).

**Results:**

On the PM testing set, nnDetPM achieved an average sensitivity of 70.2% and an average precision (at 0.1 intersection over union) of 78.1%, whereas nnDetClin fell far behind at 19.8% average sensitivity and 25.5% average precision, indicating a substantial impact of the domain shift from clinical to PM CT data. Further inspection of the results showed that the main factors of this domain shift were the position of the arms and the presence of medical ware in the image acquisition area of the PMCT scans.

**Conclusion:**

The performance of nnDetPM, with an average sensitivity of 70.2%, was notable and comparable to that of radiologists. However, more advanced techniques must be explored to decide if DL models can overcome the domain shift factors.

**Supplementary Information:**

The online version contains supplementary material available at 10.1007/s00414-025-03669-x.

## Introduction

In the University Center of Legal Medicine Lausanne-Geneva (CURML), rib fractures are found in about half of the postmortem (PM) radiological examinations. A high number of bodies underwent unsuccessful cardiopulmonary resuscitation, which produces rib fractures 4 times out of 5 [1]. Rib fractures can also be generated by fatal blunt chest trauma in traffic accidents or in falls from great heights. Additionally, many cadavers present healing and old rib fractures, which reflect the subject’s health history. In all cases, all bone fractures need to be systematically listed and classified in the forensic reports, as each of them could provide insights about the cause and manner of death. However, rib fracture detection in clinical CT imaging and in PMCT imaging remains a complex, time-consuming task, in which radiologists detect rib fractures with an average sensitivity of 75.6% [2, 3].

Deep Learning (DL) solutions have already been explored for rib fracture classification, detection and segmentation in clinical CT imaging [4]. The performance of radiologists using these models can be enhanced considerably, with an increase of their sensitivity of up to 20 percentage points and an important reduction of their time of analysis [5–20].

While the majority of researchers developed their models based on the U-Net [21], an image segmentation model, in this study we have opted for nnDetection [22], an object detection model that automatically adapts to arbitrary medical datasets.

To the best of our knowledge, only two studies exist in which a DL model was used to analyse rib fractures in PMCT scans [23, 24]. In both studies, Ibanez et al. developed PMCT classification models that achieved accuracies higher than 90%. However, there is no evidence that any research has been done on rib fracture detection or segmentation in PMCT imaging.

The main objective of this study was to determine the performance of rib fracture detection in PMCT data with nnDetection. Secondarily, we used the same model on clinical data to identify the sources of domain shift from clinical to PM CT imaging.

## Methods

### Study design

This is a feasibility study to evaluate the performance of nnDetection [22], a DL object detection model, on PMCT data of our centre, the CURML. We collected the PMCT scans retrospectively, and acute and old rib fractures were annotated by an experienced radiologist. The model was designed to be used by radiologists as a Computer-Aided Detection (CADe) tool for rib fracture detection. Additionally, a dataset of clinical CT scans with rib fracture annotation was used to train another instance of the DL model.

### Data

#### The PMRF dataset

**Fig. 1:**
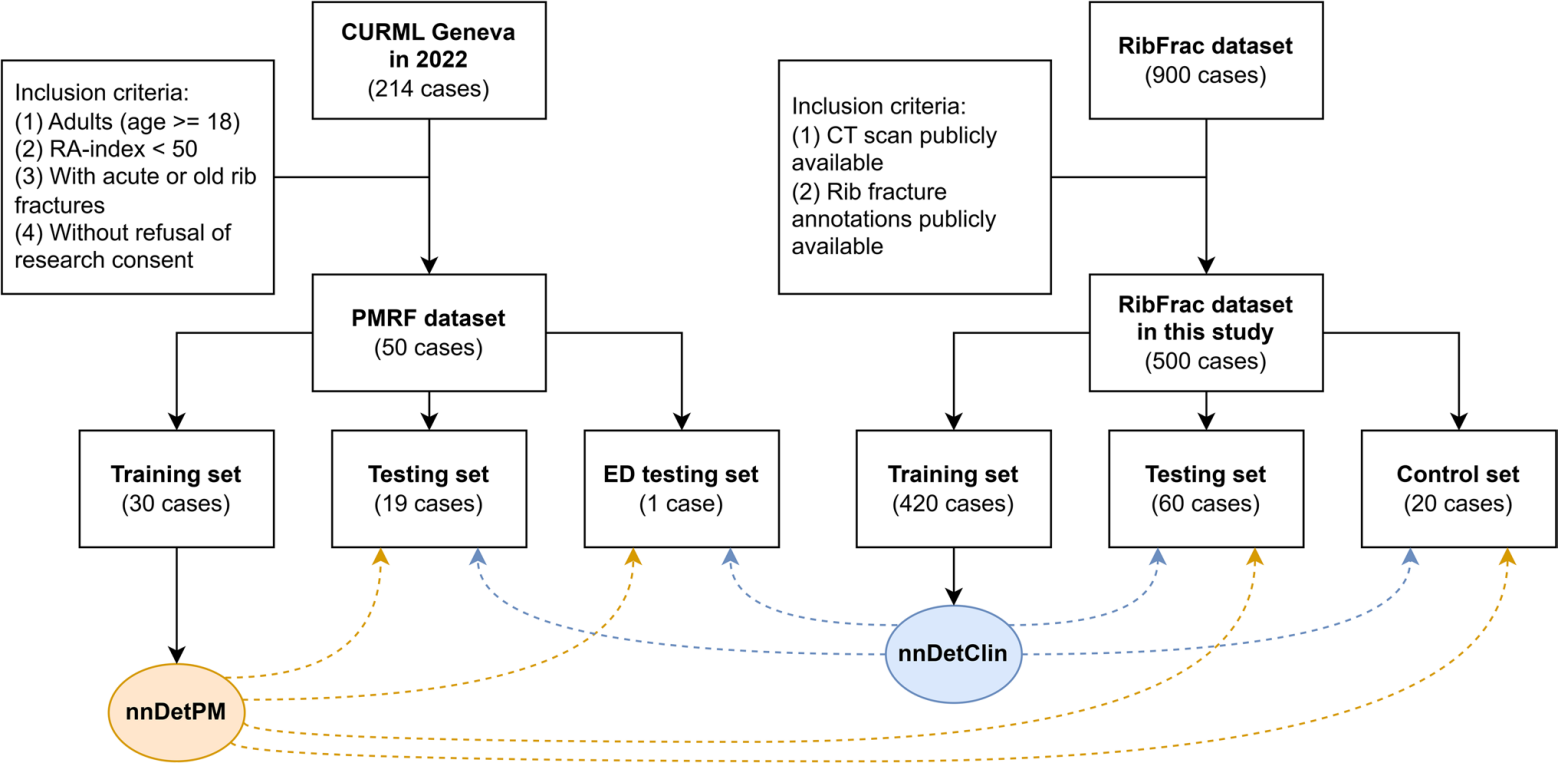
Flowchart of the criteria to create the datasets used in this study, and of the sets used to train and evaluate the models. Orange dashed arrows: evaluation of nnDetPM, blue dashed arrows: evaluation of nnDetClin

The Postmortem Rib Fracture (PMRF) dataset included a total of 50 PMCT scans (24% women; age: mean 61, range 19–96 years), acquired in 2022 from consecutive cases examined in our centre, at the site of Geneva. The cases were selected only if they fulfilled the following inclusion criteria: adult subjects (≥ 18 years) who underwent native PMCT, with low (< 50) radiological alteration (RA) index [25], and with presence of rib fractures, acute or old, according to the forensic radiology reports. As shown in Fig. 1, the dataset was divided in three sets: a training set of 30 scans, a testing set of 19 scans, and an additional testing set of only one scan of a case with a significantly larger number of lesions, which we referred to as the extensive damage (ED) testing set.

The selected native PMCT scans were produced with a LightSpeed VCT 64 scanner (GE Healthcare, IL, USA), using 120 kVp, 400 mA and 1.25 mm of slice thickness (Table 1). Only the BONE + reconstruction series of the thorax-abdomen-pelvis area were extracted from the full PMCT scans for this study. The data was kept in DICOM format after anonymisation. The rib fractures in the PMCT scans were manually annotated using 3D Slicer software (https://www.slicer.org/), and the annotated PMCT scans were stored in NRRD format.

**Table 1:** Acquisition parameters of the RibFrac and PMRF datasets

Dataset	kVp	mAs	Pitch	Collimation (mm)	Slice thickness (mm)	Reconstruction kernel
RibFrac	120	100–200	0.75–1.5	1–1.25	1–1.25	Bone or medium sharp
PMRF	120	400	1.375	0.625	1.25	BONE +

kVp: peak kilovoltage, mAs: milliampere-seconds

The annotation of PMRF was performed following a human-in-the-loop procedure. First, one radiologist with 12 years of experience (of which 5 are in forensic radiology) performed the voxel-labelling of the PMCT scans following the forensic radiology reports of each PM case, and classified each rib fracture into one of two categories: acute or old. Rib fractures in the process of healing showing features of callus development were classified as old. A first version of nnDetPM was trained with and evaluated on all 50 PMCT scans to localise rib fractures that were missing from the forensic radiology reports. A final revision of each PMCT scan was performed to complete the ground truth annotation of PMRF with all rib fractures that were missed by the first version of nnDetPM.

**Fig. 2:**
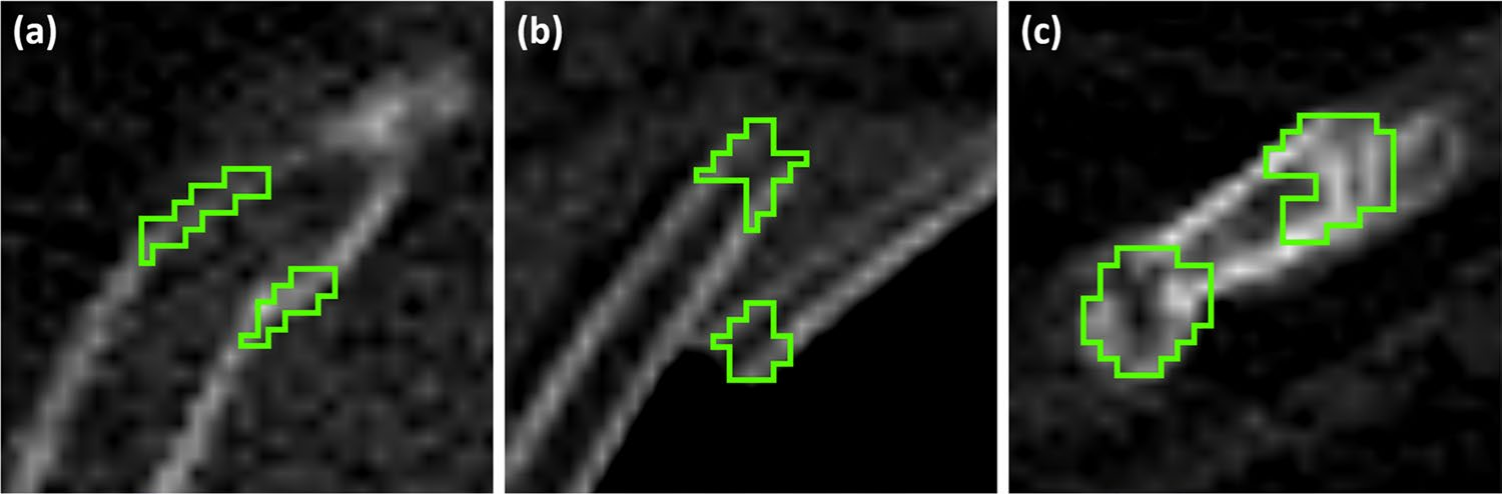
Examples of rib fracture annotations in PMRF. Green: annotation. (**a**) An angular rib fracture. (**b**) A displaced rib fracture. (**c**) Two old rib fractures

In all annotations of PMRF, the radiologist labelled only the voxels corresponding to an anomaly of the cortical bone, such as an interruption or an angulation (Fig. 2).

#### The RibFrac dataset

The RibFrac dataset was collected and annotated to be publicly released as part of an official challenge at MICCAI 2020. The challenge was divided in two tasks: instance segmentation of rib fractures, evaluated as an object detection task, and classification of rib fractures [9, 26].

The full dataset consisted of 900 CT scans (36% women; age: mean 55, range 21–94 years). However, only 660 CT scans were made publicly available, of which 420 were designed for training the models. Then, 80 CT scans were included for validation, and the remaining 160 were shared without their annotations, as they were meant for model evaluation only.

The CT scanners used to acquire the data were a Revolution CT (GE Healthcare, WI, USA) and a Somatom Definition Flash (Siemens Healthcare, Forchheim, Germany). Both scanners were set to 120 kVp, 100–200 mAs and 1–1.25 slice thickness (Table 1).

A total of five radiologists, ranging from 3 to 20 years of experience, participated in the voxel-level annotation of the CT scans. Each rib fracture instance was also classified into one out of five categories: displaced, non-displaced, buckle (angular), segmental or undefined. The last category was only assigned to ambiguous lesions for which there was no consensus among the experts.

**Fig. 3:**
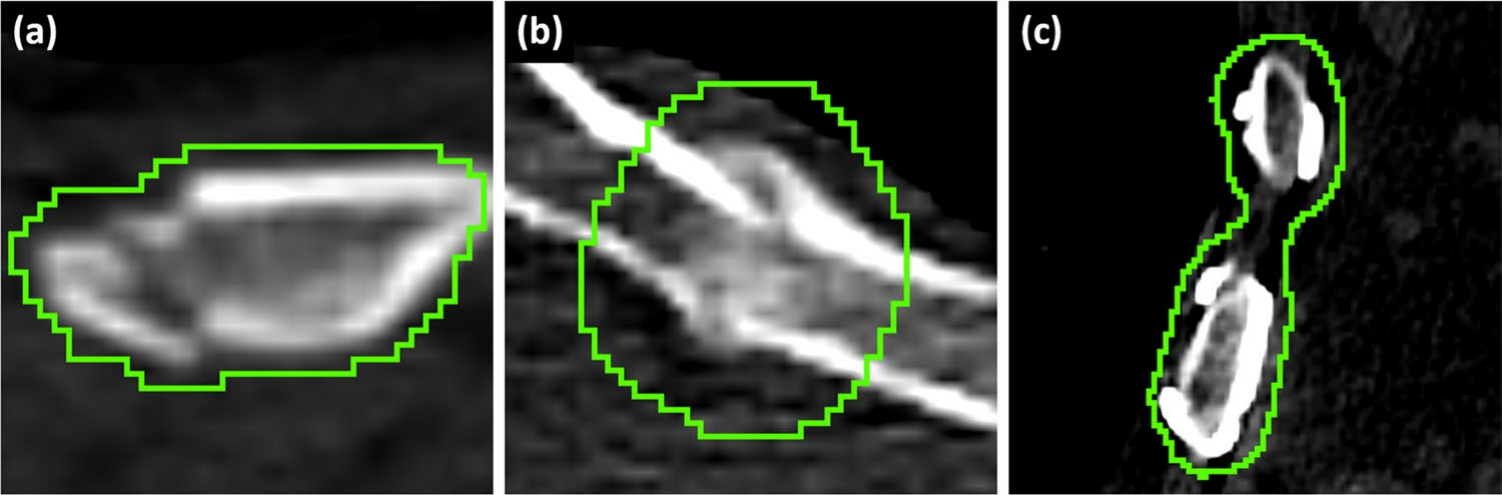
Examples of rib fracture annotations in RibFrac. Green: annotation. (**a**) A non-displaced rib fracture. (**b**) A healing rib fracture. (**c**) Old rib fractures with surgical stabilisation, annotated together

In RibFrac, rib fractures were usually annotated with a generous margin of voxels around the lesion, including healthy cortical bone and soft tissue. In some cases, rib fractures on adjacent ribs were annotated with a single instance (Fig. 3).

In this study, only the 500 CT scans of the training and validation sets with available annotations were used (Fig. 1). In particular, the 420 CT scans of the training set were used for model development, with cross-validation, and the remaining 80 CT scans for model evaluation. However, since this set of 80 CT scans contains control cases, we decided to further split it into a testing set, consisting of 60 CT scans of cases with at least one rib fracture, and a control set, made of 20 CT scans of subjects without rib fractures.

### Model

Due to its simplicity of use and great adaptability to arbitrary medical datasets, the chosen model for this study was nnDetection [22], an object detection model based on Retina U-Net, an extension of the RetinaNet [27] that can be trained with instance segmentation labels. As an object detection model, nnDetection can be trained to determine the position and size of one or more objects in the input data. In this study, the targets given to the model are groups of voxels corresponding to rib fractures. Therefore, the expected output of nnDetection is a collection of 3D bounding boxes surrounding these rib fractures.

For this study, the default configuration of nnDetection was kept unchanged: 50 epochs with 10 additional stochastic weight averaging epochs, with initial learning rate of 0.01 and stochastic gradient descent momentum of 0.9.

### Experiments

Two instances of nnDetection were trained: nnDetClin was trained on RibFrac, while nnDetPM was trained on PMRF. As represented in Fig. 1, both models were applied to all testing sets. Finally, an additional instance of nnDetection, denoted nnDetPM_CLASS_, was trained on PMRF to detect acute and old rib fractures separately.

### Training environment

During the fivefold cross-validation, each fold was trained on six 64 GB CPUs and an 11 GB Turing RTX 2080 Ti, at a speed of approximately 40 min per epoch.

## Results

### PMRF dataset annotation

A total of 698 rib fractures were annotated in the 50 PMCT scans from PMRF. Excluding the ED case with 79 rib fractures, PMRF was split into a training set and a testing set, with a ratio of annotations per PMCT scan of 12.6 and 12.7, respectively. On the other hand, the training and testing sets of RibFrac had annotations at a ratio of 9.5 and 7.3 rib fractures per scan, respectively (Table 2).

**Table 2:** Number of CT scans and rib fracture annotations in the RibFrac and PMRF datasets

Dataset (n scans)	Set (n scans)	RF	RF/scan
RibFrac (500)	Training (420)	3987	9.5
	Testing (60)	435	7.3
	Control (20)	0	0.0
PMRF (50)	Training (30)	378	12.6
	Testing (19)	241	12.7
	Testing ED (1)	79	79.0

RF: rib fracture annotations

Out of the 698 ground truth rib fracture annotations, 619 were produced in the first round of annotations, following the forensic radiology reports, while 79 were included in the final revision of the PMCT annotations, out of which 34 were proposed by the first version of nnDetPM, and the remaining 45 were neither listed in the reports nor found by the first version of nnDetPM.

**Fig. 4:**
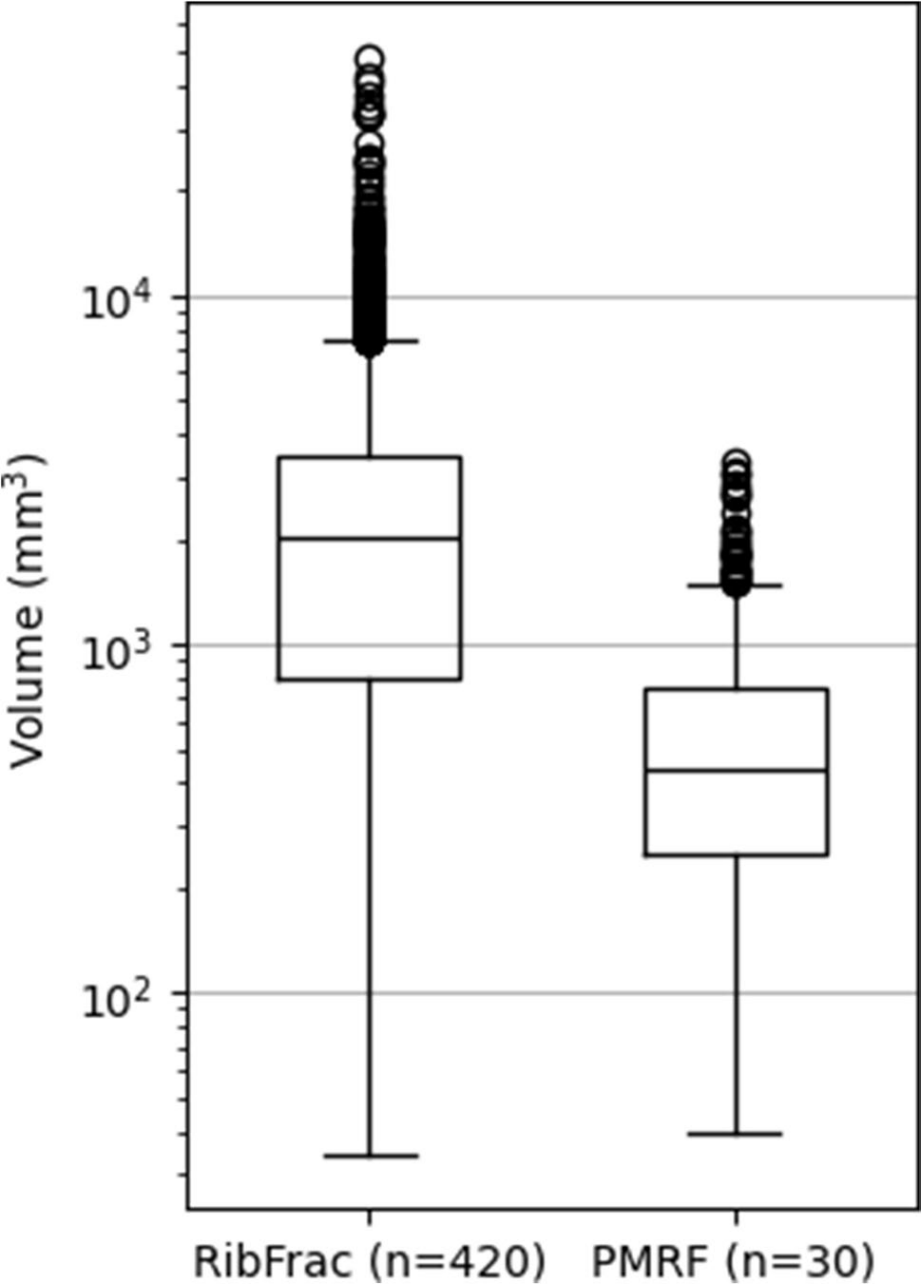
Volume of annotations of the RibFrac and PMRF training sets (n: number of CT scans)

Due to the different annotation guidelines of each dataset, the median volume of an annotation in the RibFrac training set was 2013.81 mm^3^, while in the PMRF training set the median volume was 437.74 mm^3^. Figure 4 shows the distributions of these volumes, with the first and third quartiles equal to 793.14 and 3439.71 mm^3^ in the RibFrac training set, while in the PMRF training set these quartiles were 248.67 and 755.55 mm^3^.

### Rib fracture detection on RibFrac

The application of nnDetClin on RibFrac reached an average sensitivity of 77.0%. Table 3 shows the detailed performance results of nnDetClin together with those of other published models on different sets of the RibFrac dataset.

**Table 3:** Rib fracture detection on different RibFrac evaluation sets

Model	RibFrac set (n scans)	IoU thr	FROC sensitivities at FPPS						Max Sens
			0.5	1	2	4	8	Avg	
FracNet [9]	Val (60)	0.2	55.6	67.8	78.9	89.7	92.2	76.8	92.2
FasterRib [28]	Val + Cont (80)	0.5	-	-	-	-	-	-	95.0
Comp Att Res U-Net [29]	Val + Cont (80)	-	-	-	-	-	-	-	81.7
FracNet + [26]	Test (160)	0.2	75.7	79.2	84.3	90.8	91.4	84.3	92.2
Dual Att RetinaNet [30]	Val + Cont (80)	-	76.4	83.5	86.9	90.2	91.3	85.7	91.3
nnDetClin (ours)	Val (60)	0.1	61.1	71.3	79.1	85.3	88.3	77.0	88.3

IoU thr: Intersection over Union threshold, FROC: Free-response Receiver Operating Characteristic, FPPS: False Positives Per Scan, Sens: sensitivity, Val: validation, Cont: control

As shown in Table 4, nnDetClin achieved the best performance at 77% average sensitivity, while nnDetPM obtained moderate results, with an average sensitivity of 61.5%.

**Table 4:** Rib fracture detection performance of nnDetection models on the RibFrac testing set. Best results are shown in bold

Model	AP (IoU 0.1) (%)	mAP (IoU 0.05, 0.1, 0.5) (%)	Avg Sens (%)
nnDetClin	**80.5**	**62.1**	**77.0**
nnDetPM	66.1	46.7	61.5

AP: Average Precision, mAP: mean Average Precision, IoU: Intersection over Union, Avg: average, Sens: sensitivity

### Rib fracture detection on PMRF

When applied to the PMCT scans of the PMRF testing set, nnDetPM achieved the best rib fracture detection results, with an average sensitivity of 70.2%. The clinical model, nnDetClin, on the other hand, reached only 19.8% average sensitivity (Table 5).

**Table 5:** Rib fracture detection performance of nnDetection models on the PMRF testing set. Best results are shown in bold

Model	AP (IoU 0.1) (%)	mAP (IoU 0.05, 0.1, 0.5) (%)	Avg Sens (%)
nnDetClin	25.5	17.9	19.8
nnDetPM	**78.1**	**66.1**	**70.2**

AP: Average Precision, mAP: mean Average Precision, IoU: Intersection over Union, Avg: average, Sens: sensitivity

**Fig. 5:**
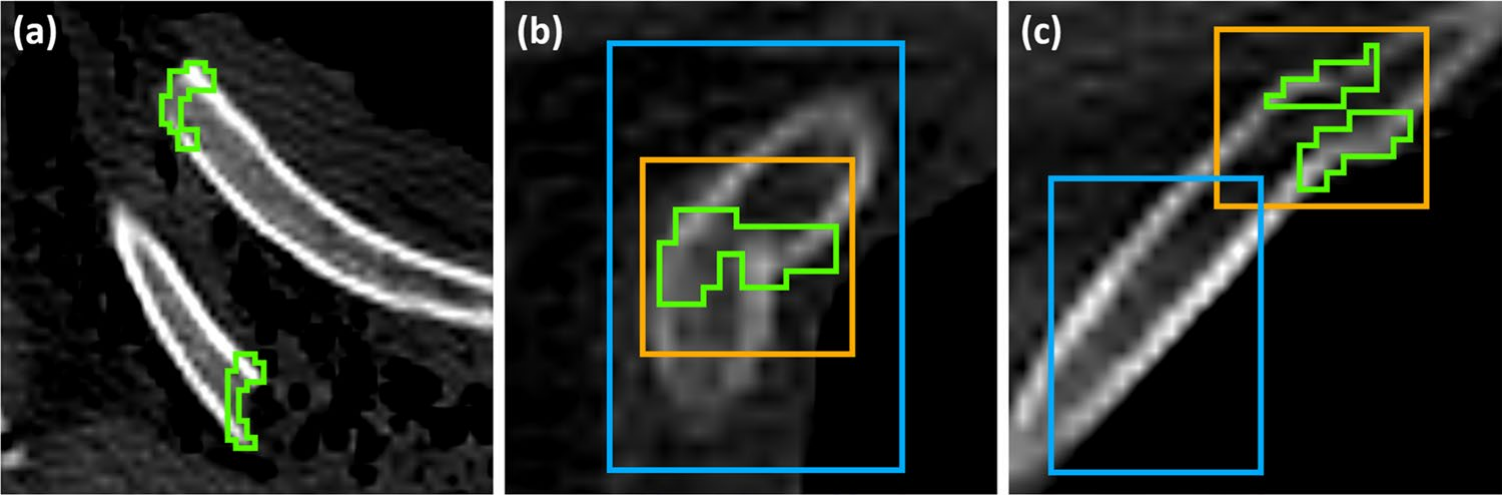
Examples of model results on PMRF. Green: annotation, blue: nnDetClin, orange: nnDetPM. (**a**) FN by both models on a displaced rib fracture. (**b**) TP by both models on an angular rib fracture. (**c**) TP by nnDetPM, FP and FN by nnDetClin on an anterior rib fracture

**Fig. 6:**
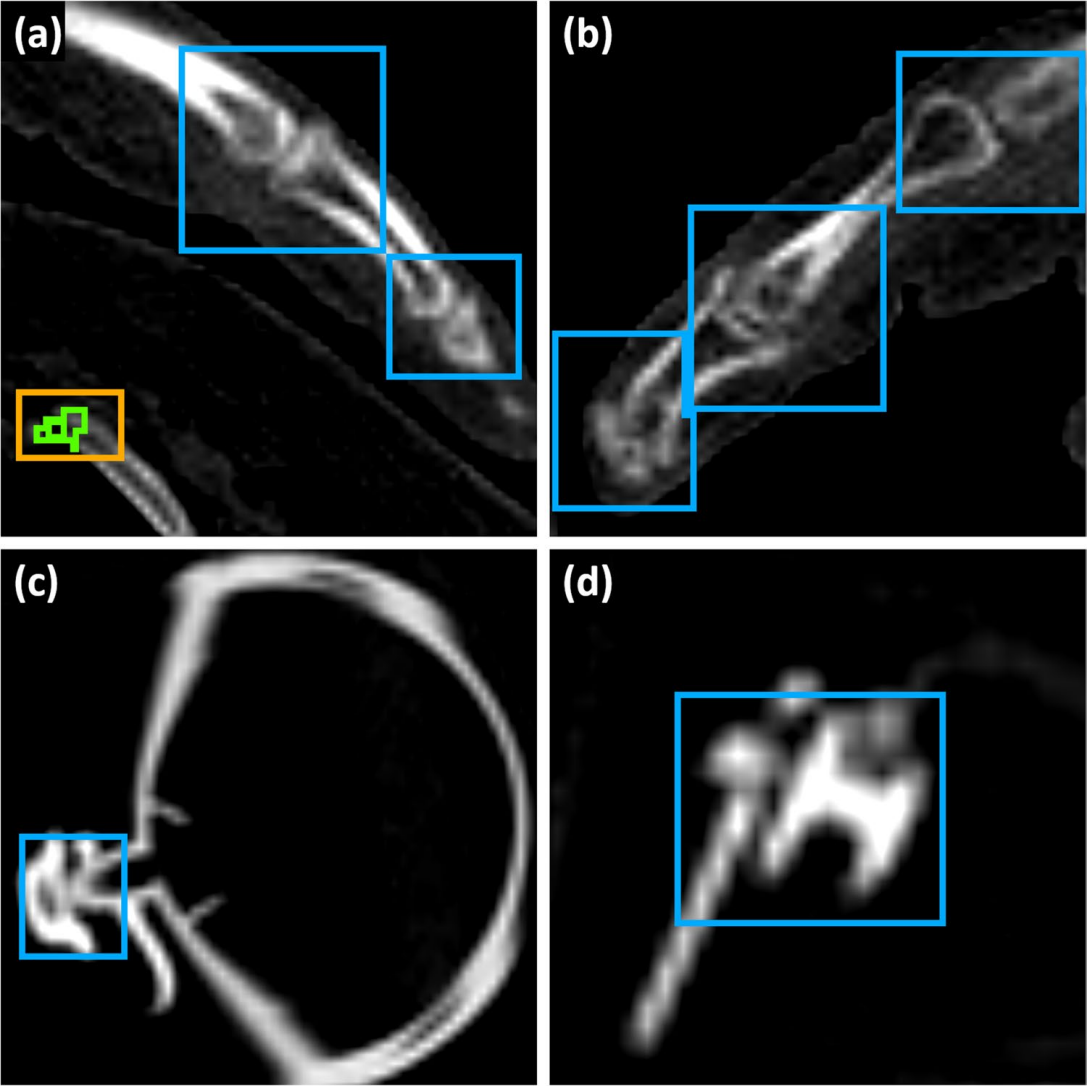
Examples of FP by nnDetClin on PMRF. Green: annotation, blue: nnDetClin, orange: nnDetPM. (**a**) On phalanges, and a TP of nnDetPM on an anterior rib fracture. (**b**) On phalanges. (**c**) On breathing ware. (**d**) On a zipper

Further inspection of model detection boxes showed that, while nnDetClin missed the majority of rib fractures in the anterior, posterior and para-vertebral areas, it detected old rib fractures slightly better than nnDetPM, with sensitivities of 73% and 54%, respectively (see Fig. 5 for some examples of true positives (TP), as well as false positives (FP) and false negatives (FN)). Moreover, the majority of FP produced by nnDetClin on PMRF corresponds to bone junctions between phalanges, followed by diverse medical ware, such as ECG monitoring cables, endotracheal tubes and zippers (Fig. 6).

### Rib fracture classification on PMRF

Out of the 378 rib fractures in the PMRF training set, 244 (65%) were annotated as acute, and 134 (35%) were labelled as old. In the testing set, the 241 rib fracture annotations consisted of 204 (85%) acute fractures and 37 (15%) old fractures.

An additional instance of nnDetection was trained to produce outputs with a fracture type classification: acute (label 0) or old (label 1). While nnDetPM_CLASS_ detected acute rib fractures with an average sensitivity of 69.9%, which was comparable to the 70.2% of nnDetPM, its performance with old rib fracture detection was much lower, at an average sensitivity of 51.5% (Table 6).

**Table 6:** Rib fracture detection performance of nnDetPM_CLASS_ on the PMRF testing set. Best results are shown in bold

Rib fracture type	AP (IoU 0.1) (%)	mAP (IoU 0.05, 0.1, 0.5) (%)	Avg Sens (%)
Acute	**76.7**	**64.5**	**69.9**
Old	49.6	44.4	51.5

AP: Average Precision, mAP: mean Average Precision, IoU: Intersection over Union, Avg: average, Sens: sensitivity

In fact, the classification model nnDetPM_CLASS_ generated practically the same number of TP and FN for both acute and old rib fractures as the anomaly-detection model nnDetPM. Indeed, both nnDetPM and nnDetPM_CLASS_ identified only about half of the 37 old rib fractures in the testing set, while the rest were missed.

### FP analysis on all testing sets

Although nnDetClin obtained better results when applied to the RibFrac testing set, it produced slightly more FP than nnDetPM, with FPPS rates at 3.15 and 2.68 respectively (Table 7). In fact, nnDetPM generated less FP on the rest of testing sets, including the RibFrac control testing set, where nnDetClin outputed an average of 1.95 FPPS and nnDetPM is at 0.75 FPPS.

**Table 7:** Rib fracture FP analysis of nnDetection models (at thresholds IoU 0.1 and confidence 0.5) on the RibFrac and PMRF testing sets. Best results are shown in bold

Dataset	Set (n scans)	Model	FP	FPPS
RibFrac	Testing (60)	nnDetClin	189	3.15
		nnDetPM	**161**	**2.68**
	Control (20)	nnDetClin	39	1.95
		nnDetPM	**15**	**0.75**
PMRF	Testing (19)	nnDetClin	118	6.21
		nnDetPM	**33**	**1.74**
	Testing ED (1)	nnDetClin	16	16
		nnDetPM	**0**	**0**

FP: False Positive, FPPS: False Positives Per Scan, IoU: Intersection over Union

### Rib fracture detection on PMRF ED

**Fig. 7:**
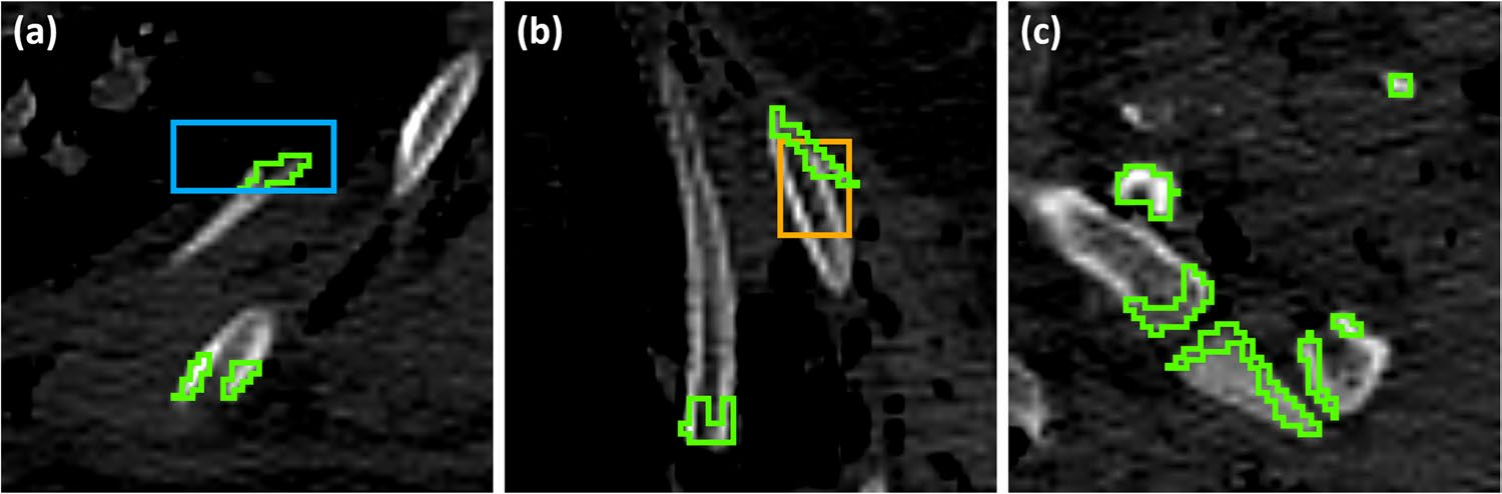
Examples of model results on the ED testing set of PMRF. Green: annotation, blue: nnDetClin, orange: nnDetPM. (**a**) FN by both models and TP by nnDetClin on a displaced rib fracture. (**b**) FN by both models and TP by nnDetPM on a displaced rib fracture. (**c**) FN by both models on a comminuted rib fracture

Both nnDetClin and nnDetPM performed poorly on the ED testing set, which had 79 rib fracture annotations. While nnDetPM found 18 rib fractures and produces no FP, nnDetClin only finds 4 rib fractures and produced 16 FP, half of which were located in the costochondral junction. The models performed the worst on rib fractures with a large distance of displacement (24 FN by each model), followed by rib fractures in the para-vertebral zone and comminuted rib fractures (Fig. 7).

## Discussion

In this study, we collected and annotated 50 PMCT scans, with which we trained a DL model that achieved 70.2% sensitivity on rib fracture detection. With a larger dataset and further development, we believe that this model could be used by radiologists as a CADe tool and enhance rib fracture detection in forensic investigations.

### Main findings

Besides the nnDetPM model, the instance of nnDetection trained on our PMCT scans, we trained another instance of nnDetection on RibFrac, that we denoted as nnDetClin. Each instance performed well on its respective dataset: nnDetClin achieved an average sensitivity of 77.0% on RibFrac and nnDetPM scored 70.2% on PMRF. However, nnDetClin obtained poor results when applied to PMRF, reaching only 19.8% average sensitivity. Conversely, nnDetPM achieved rather good results when applied to RibFrac, with an average sensitivity of 61.5%, suggesting that the domain shift had less impact from PM to clinical CT data.

Despite its adaptability, nnDetection's autoconfiguration processes might not be optimal in certain cases, making it underperform against other models. As shown in Table 3, while nnDetection performed similarly to FracNet [9] in terms of average sensitivity and average FPPS, FracNet achieved a higher maximum sensitivity of 92.2% at 8 FPPS against nnDetClin’s 88.3%. The approach followed to develop FracNet was to use a customised 3D U-Net [21] with additional data processing steps to optimise results. In particular, CT scans were first preprocessed to extract bone areas by applying a series of morphological operations, followed by clipping voxel values to the bone window. The 3D U-Net read the normalised data in patches of 64 × 64 × 64 voxels and outputed rib fracture segmentations for each patch, which were then reassembled by keeping maximum values in overlapping regions. Afterwards, the raw segmentation was postprocessed to reduce FP, with techniques such as removing small connected components. Yang et al. extended FracNet to FracNet + by integrating a rib segmentation model to preprocess data [26]. This step enhanced the accuracy and efficiency of the sliding-window approach to detect rib fractures. Additionally, FracNet + used a STU-Net [31], a U-Net based model pretrained with the TotalSegmentator dataset [32]. To adapt the STU-Net to rib fracture detection, the full model was fine-tuned. As reported in Table 3, this improved version of FracNet reached an average sensitivity of 84.3% and reduced the FPPS to 4.22 when applied on the RibFrac testing set (160 CT scans). Another interesting result is that of [30], where a RetinaNet architecture with a Dual Attention lead to the highest average sensitivity, 85.7%, with considerably high values of sensitivity at low levels of FPPS on the RibFrac validation set (60 CT scans and 20 control CT scans).

### Domain shift findings

The revision of FP and FN produced by nnDetClin on PMRF led us to the identification of a number of factors that could explain the clinical to PM CT data domain shift, and, conversely, the analysis of FP and FN of nnDetPM on RibFrac showed the impact of the domain shift from PM to clinical CT imaging. First, when taking a clinical CT scan of the thorax-abdomen-pelvis area, the patient holds their arms above their head, which is not the case in the morgue, where most bodies have their arms and hands on their thoraxes or abdomens due to rigor mortis. As illustrated in the examples in Fig. 6, nnDetClin identified phalanges as rib fractures, as these structures are similar to the target anomalies that this model is trained to detect. Second, a significant amount of PMCT scans are acquired with bodies coming straight from an emergency intervention, with ECG cables, tubes and other medical ware present in the CT scanner image acquisition area. Some of these objects were mistaken for rib fractures by nnDetClin, as it can be seen in Fig. 6. Similarly, the RibFrac dataset contained some CT scans of patients with surgical stabilisation on old rib fractures, which nnDetPM failed to detect. Third, even if cases with RA-index > 50 were excluded from PMRF, the presence of air and bubbles in the soft tissue around ribs may have an influence as well on the performance of nnDetClin. Overall, the impact of the domain shift from clinical to PM CT data appears to be larger than from PM to clinical CT imaging. While there might be CT scan preprocessing algorithms or even robust DL models to overcome these factors, we hypothesize that the clinical to PM CT imaging domain shift is too large, and that a DL model exclusively trained on PMCT data can generalise better.

Another factor of domain shift is the difference in acquisition parameters between RibFrac and PMRF, especially the radiation dose and the reconstruction kernel. While we believe that small differences in acquisition parameters might not that impactful to the performance of a DL model, research has shown that the change of sharpness of the reconstruction kernel leads to unstable predictions [33].

Finally, we also observed that the size of annotations had a strong repercussion on the DL model’s output. Indeed, nnDetClin learned to make detections of large volume, which could be considered to be FP if the IoU with the ground truth was below the predefined threshold. This is, in fact, not a factor of domain shift, as it is not inherent of the CT data, but a product of the different annotation guidelines. To avoid reannotating the CT scans, the voxel segmentation labels could be transformed to 3D bounding boxes, bringing the size of annotations in RibFrac and PMRF closer.

### Other findings

Lastly, in the rib fracture classification experiment, nnDetPM_CLASS_ produced approximately the same TP and FN as nnDetPM with extra labels to classify the outputs as acute or old rib fractures. While this classification model identified about 70% of acute rib fractures on PMRF, it could only find half of the old rib fractures.

Also, nnDetPM produced less FP than nnDetClin in all testing sets. Lastly, both nnDetPM and nnDetClin underperformed on the ED case of PMRF, missing several rib fractures with large displacement or in the para-vertebral area.

### Limitations and future research

The main limitation of this study is the scarce number of cases in PMRF, which reduced the significance of the results obtained in the evaluation of the models on the PMRF testing set. In addition, only half of the PMCT scans contained old rib fractures, and complex rib fractures (such as comminuted, para-vertebral or displaced rib fractures with a large distance of separation) were only present in a few PMCT scans, among which the ED testing set case. This lack of positive samples made these types of fractures more challenging to identify by the model. Lastly, since only one radiologist participated in the annotation process, the rib fracture labels of PMRF may have subjective bias.

Thus, future work will consist in the development of a larger dataset with PMCT scans from more than one centre and annotated by at least two experts. Additionally, we will explore self-supervised learning solutions, so that we can pretrain our model with unlabelled PMCT data. Indeed, such a model could learn by solving a pretext task in an unsupervised manner, and afterwards be trained to solve a specific task, from PMCT screening to lesion segmentation [34–36]. Alternatively, PMCT scans could be preprocessed with a full body segmentation model, such as TotalSegmentator [32], before training the DL model to detect and classify specific lesions.

## Conclusion

The nnDetection model trained with PMCT scans reached notable rib fracture detection performance, achieving an average sensitivity of 70.2% on PMCT scans and 61.5% on clinical CT scans. We believe that, in spite of the potential in leveraging PMCT scans to improve a model for clinical CT data, a more advanced strategy is required to determine if the domain shift from clinical to PM CT imaging can be overcome.

## Supplementary Information

Below is the link to the electronic supplementary material.

Supplementary file1 (DOCX 19 KB)

## Data Availability

The PMRF dataset generated and analysed in this study can be available from the corresponding author on reasonable request.
