# Supplementary material for: Automatic rib fracture detection on postmortem CT data using deep learning
Source: Int J Legal Med. 2025 Dec 4;140(2):857–66. doi: 10.1007/s00414-025-03669-x (PMC12957061; doi:10.1007/s00414-025-03669-x)
Supplement: Supplementary file 1 — Supplementary file1 (DOCX 19 KB) [file 414_2025_3669_MOESM1_ESM.docx]

# Supplementary material

In this document, the reader will find a short literature review on other solutions developed for rib fracture analysis in CT and in postmortem (PM) CT data.

## Review

Rib fracture classification, detection and segmentation in CT data with Deep Learning (DL) solutions has already been explored. In many studies, researchers chose a common DL model, the U-Net [1], for rib fracture detection and rib fracture segmentation. For instance, Jin et al. used a customised 3D U-Net that detected rib fractures at a maximum sensitivity of 92.9%, with an average number of false positives per scan (FPPS) of 5.27 [2]. An extension of this model, the nnU-Net [3], included automatic configuration of hyperparameters for optimal adaptation to arbitrary medical datasets. Thanks to this new method, the developers of nnU-Net won the Medical Segmentation Decathlon challenge at the Medical Image Computing and Computer Assisted Intervention (MICCAI) in 2018, beating other teams using U-Net-based models [4]. Finally, a model with a hybrid architecture, nnDetection [5], performed better than improved nnU-Nets on rib fracture detection, with an Average Precision (AP) of 76.5% against the 70.0% obtained by nnU-Net Plus, at an Intersection over Union (IoU) threshold of 0.1.

Besides RibFrac, there are several other private CT scan datasets on which DL has been trained to perform rib fracture detection. Li et al. collected an astounding total of 14241 CT scans and used them for the development of a DL model, which detected acute rib fractures with a cascade-based design [6]. First, an initial 2D object detection model selected axial slices that were suspected to contain rib fractures, followed by a 3D ResNet 50 that found rib fracture candidates within the adjacent axial slices, eliminating FP from the first step. An additional 3D U-Net segmented ribs so that the model could also provide a rib level (1 to 12) for all rib fracture detections. Zhou et al. developed a Faster R-CNN model to detect rib fractures in 2D axial slices, followed by an aggregation of 2D bounding boxes to report results as 3D bounding boxes [7]. The model was trained to distinguish among acute, healing and old rib fractures. Posteriorly, in another research project by the same team, the Faster R-CNN model was extended to integrate clinical information of the patient in text form, in addition to the 2D axial slices of each corresponding CT scan [8]. This cross-modal architecture proved to outperform the single-modal instance of Faster R-CNN. Finally, in another study, the team used a RetinaNet with an improved reconstruction algorithm to rank ribs and include rib cage side (left, right) and rib level (1 to 12) in the report of rib fracture detections [9]. In these four studies, the sensitivity and precision of radiologists while using the DL models as CADe tools was proven to increase with respect from their usual performance, while also reducing the time of analysis [6-9].

Only two studies have been published on DL models for rib fracture analysis on PMCT scan data. In both studies, Ibanez et al. used a commercial software to preprocess each PMCT scan into a 2D image of the unfolded rib cage. In the first study, the unfolded rib cage images were used as input for a DL model that classified them into positive and negative for rib fracture presence with an accuracy of 91% and a sensitivity of 93% [10]. In the second study, the inputs of the DL model were patches of 99 x 99 pixels, which were extracted from the unfolded rib cage images [11]. The model was trained to classify the patches into positive or negative for rib fractures with an aggregated accuracy of 99.3%. Furthermore, the model also classified positive patches into rib fracture categories, such as displaced and non-displaced.

## References

[1] Ronneberger O, Fischer P, Brox T (2015) U-Net: convolutional networks for biomedical image segmentation. Medical Image Computing and Computer-Assisted Intervention (MICCAI) 9351:234-241. <https://doi.org/10.1007/978-3-319-24574-4_28>

[2] Jin L, Yang J, Kuang K et al. (2020) Deep-learning-assisted detection and segmentation of rib fractures from CT scans: Development and validation of FracNet. EBioMedicine 62:103106. <https://doi.org/10.1016/j.ebiom.2020.103106>

[3] Isensee F, Jaeger PF, Kohl SA, Petersen J, Maier-Hein KH (2021) nnU-Net: a self-configuring method for deep learning-based biomedical image segmentation. Nature methods 18(2):203-211. <https://doi.org/10.1038/s41592-020-01008-z>

[4] Antonelli M, Reinke A, Bakas S et al. (2022) The Medical Segmentation Decathlon. Nat Commun 13(4128). <https://doi.org/10.1038/s41467-022-30695-9>

[5] Baumgartner M, Jäger PF, Isensee F, Maier-Hein KH (2021) nnDetection: A Self-configuring Method for Medical Object Detection. Medical Image Computing and Computer Assisted Intervention (MICCAI) 12905. <https://doi.org/10.1007/978-3-030-87240-3_51>

[6] Li N, Wu Z, Jiang C et al. (2023) An automatic fresh rib fracture detection and positioning system using deep learning. Br J Radiol 96(1146):20221006. <https://doi.org/10.1259/bjr.20221006>

[7] Zhou QQ, Wang J, Tang W et al. (2020) Automatic detection and classification of rib fractures on thoracic CT using convolutional neural network: accuracy and feasibility. Korean J Radiol 21(7):869-879. <https://doi.org/10.3348/kjr.2019.0651>

[8] Zhou QQ, Tang W, Wang J et al. (2021) Automatic detection and classification of rib fractures based on patients' CT images and clinical information via convolutional neural network. Eur Radiol 31(6):3815-3825. <https://doi.org/10.1007/s00330-020-07418-z>

[9] Zhou QQ, Hu ZC, Tang W et al. (2022) Precise anatomical localization and classification of rib fractures on CT using a convolutional neural network. Clin Imaging 81:24-32. <https://doi.org/10.1016/j.clinimag.2021.09.010>

[10] Ibanez V, Gunz S, Erne S et al. (2022) RiFNet: Automated rib fracture detection in postmortem computed tomography. Forensic Sci Med Pathol 18(1):20-29. <https://doi.org/10.1007/s12024-021-00431-8>

[11] Ibanez V, Jucker D, Ebert LC et al. (2023) Classification of rib fracture types from postmortem computed tomography images using deep learning. Forensic Sci Med Pathol. <https://doi.org/10.1007/s12024-023-00751-x>
